# Supplementary material for: PAX6 mutation alters circadian rhythm and β cell function in mice without affecting glucose tolerance
Source: Commun Biol. 2020 Oct 30;3:628. doi: 10.1038/s42003-020-01337-x (PMC7599253; doi:10.1038/s42003-020-01337-x)
Supplement: Supplementary file 1 — Supplementary Information [file 42003_2020_1337_MOESM1_ESM.pdf]

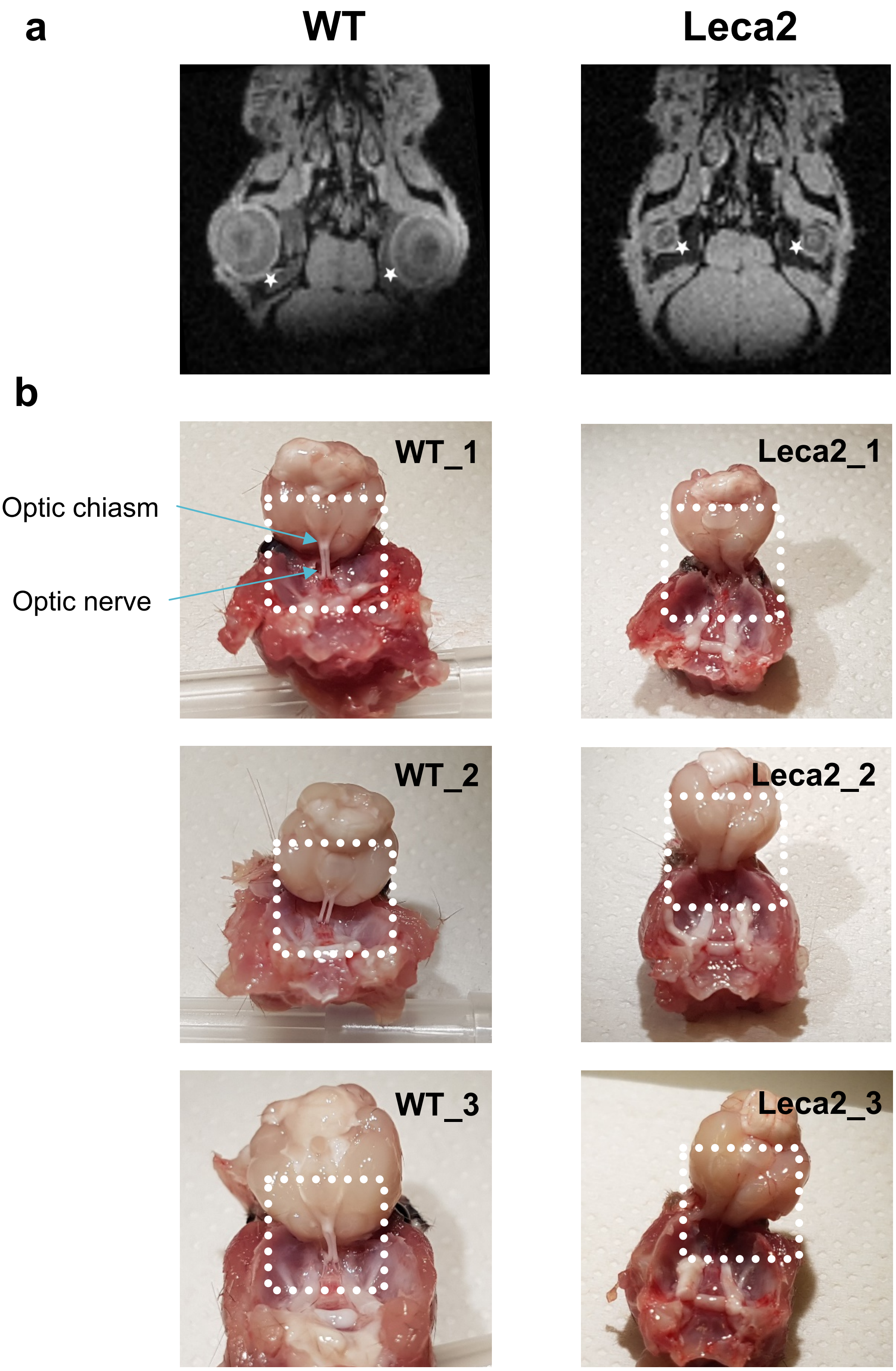

Supplementary fig 1 Lack of optic nerve and optic chiasm in homozygous Pax6^{Leca2} mice. a Representative MRI images displaying eye and eye structures in WT and mutant mice (white asterix). **b** Ventral aspect of the brain displaying presence of optic nerve and optic chiasm in the WT and lack thereof in mutants (white dotted box). 10-12-week old male mice were used for this study.

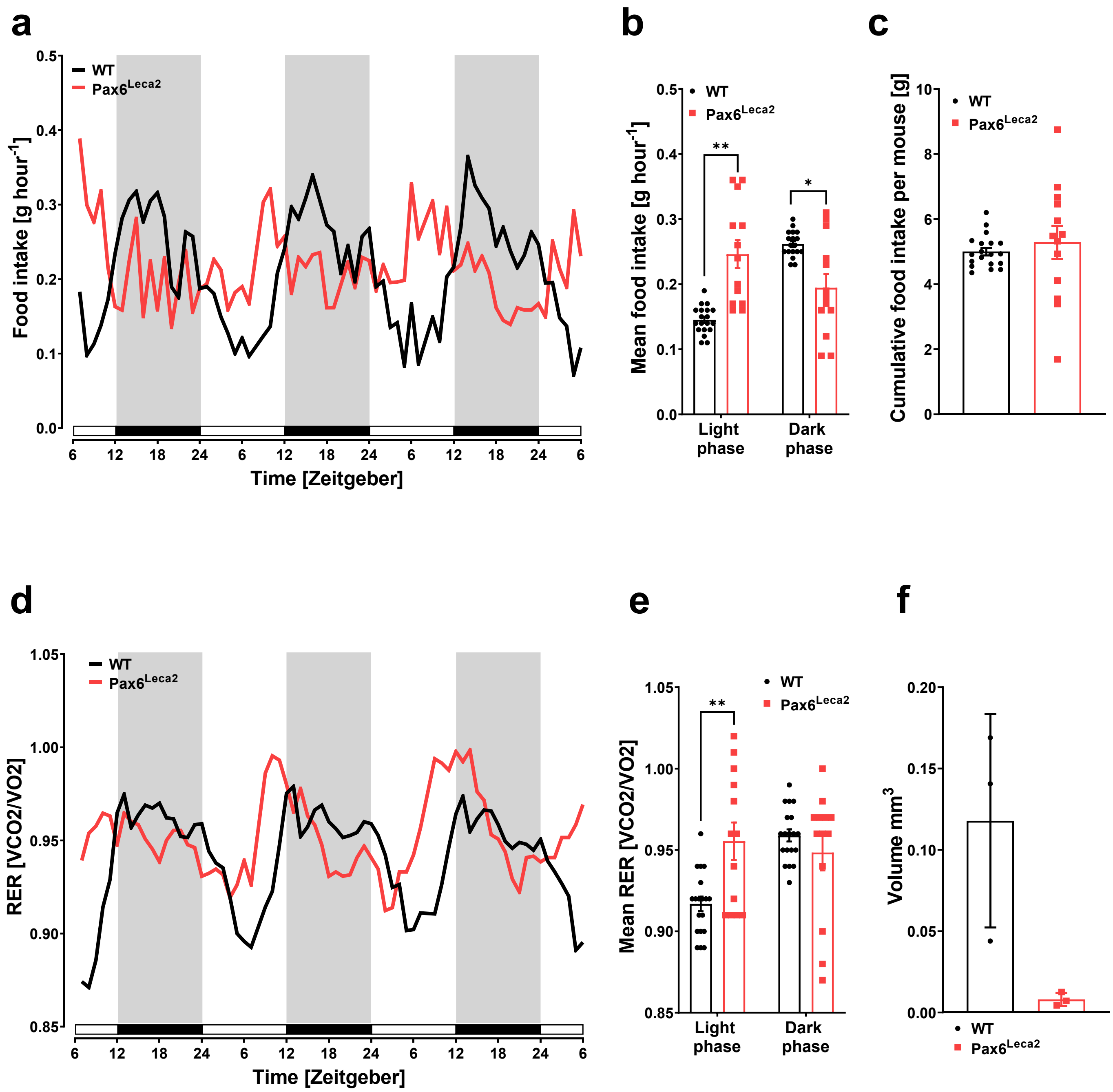

Supplementary fig 2 Changes in food intake and RER in Pax6^{Leca2} mice. a-e Indirect calorimetry measurements taken over 72 hours displaying (**a**) food intake, (**b**) average food intake (WT n=19, Leca2 n=13, **p*<0.05, ***p*<0.01 one-way Welch's ANOVA followed by Dunnett's *post hoc* test) and (**c**) cumulative food intake per mouse, (**d**) Respiratory exchange ratio (RER) and (**e**) average RER (WT n=19, Leca2 n=13, ***p*<0.01 one-way Welch's ANOVA followed by Dunnett's *post hoc* test). 14-week-old male mice were used for this study. Error bars display ±s.e.m. Grey shade and black bars depict light off and white shade and bars depict lights on. **f** Volumetric analysis of µCT images of pineal gland (n=3). 18-week old mice were used in this study. Error bars display ±s.d.

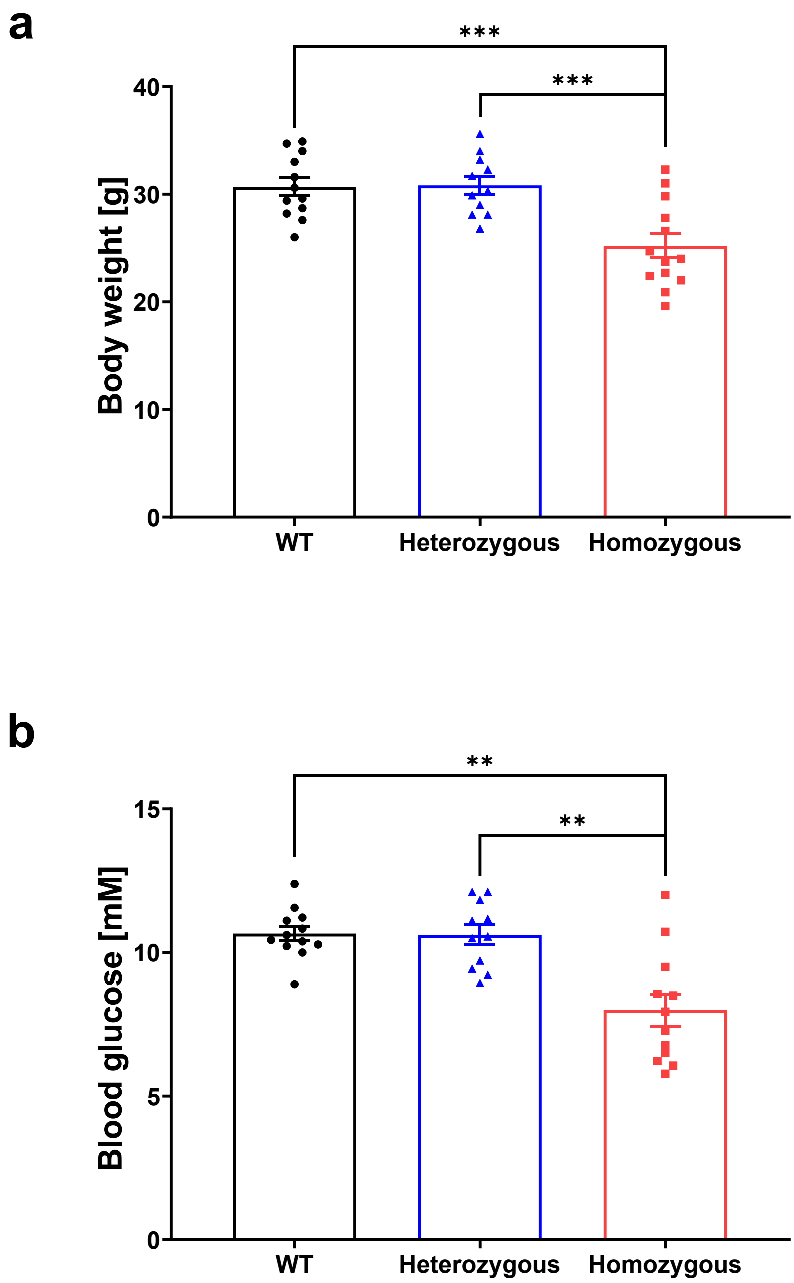

Supplementary fig 3 Heterozygous Leca2 mutants show normal blood glucose and body weight. a, b 6-hour fasted measurements of (a) body weight and (b) blood glucose levels (WT n=12, Het n=12, Hom n=13 (**a**), n=12 (**b**)). $^{**}p<0.01$, $^{***}p<0.001$ one-way or Welch's ANOVA followed by Bonferroni's or Dunnett's *post hoc* test. 12-week-old male mice were used for this study. Error bars display ±s.e.m.

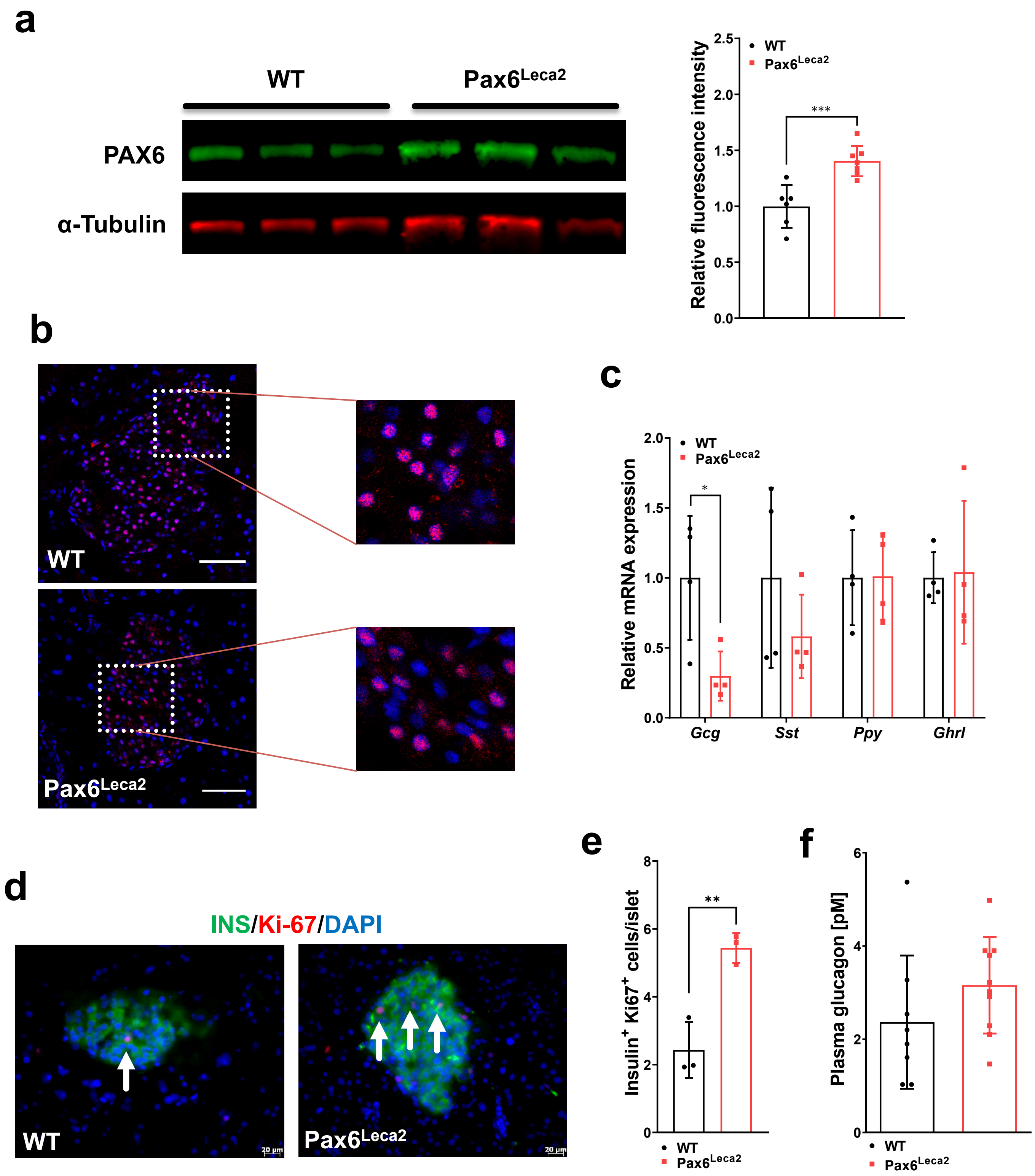

Supplementary fig 4 Leca2 mutation does not affect islet architecture. a Representative western blot images and quantification of PAX6 expression. WT n=6, Leca2 n=7. ***p<0.001 Student's t test. **b** Representative images of nuclear expression of PAX6 in pancreatic islets. **c** Relative islet mRNA expression of genes encoding hormones in 10-week-old male mice. n=4. *p<0.05 Student's t test. **d** Representative immunofluorescence images of cells positive for insulin and proliferation marker Ki-67 (arrows), and respective quantifications thereof in **e**. ~60 islets per genotype were analyzed, n=3, Scale bars, 20 μm. **p<0.01 Student's t test. **f** Plasma glucagon levels after a 6-hour fasted period. WT n=8, Leca2 n=10. Error bars display ±s.d.

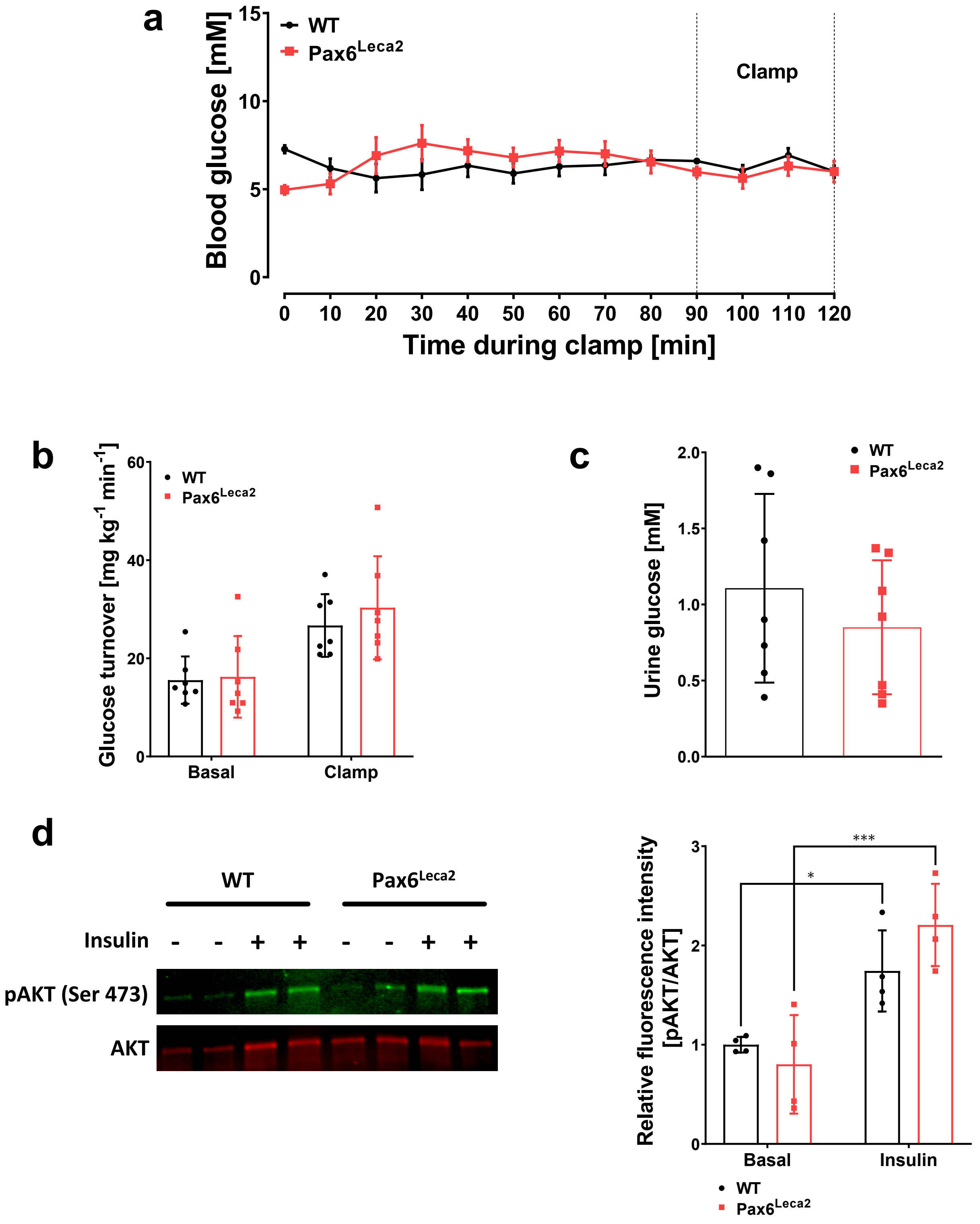

Supplementary fig 5 Pax6^{Leca2} mice do not show any change in total glucose turnover. **a-c** Measurements of (**a**) blood glucose levels, (**b**) glucose turnover and (**c**) loss of glucose via urine during a hyperinsulinemic-euglycemic clamp using 12-14-week-old male mice. n=7. **d** Representative western blot images and quantification of AKT and pAKT expression in liver samples of 14 week old mice after stimulation with PBS or insulin (1 U/kg). n=4. *p<0.05, ***p<0.001 one-way ANOVA followed by Bonferroni's *post hoc* test. Error bars display ±s.e.m. in **a**, rest ±s.d.

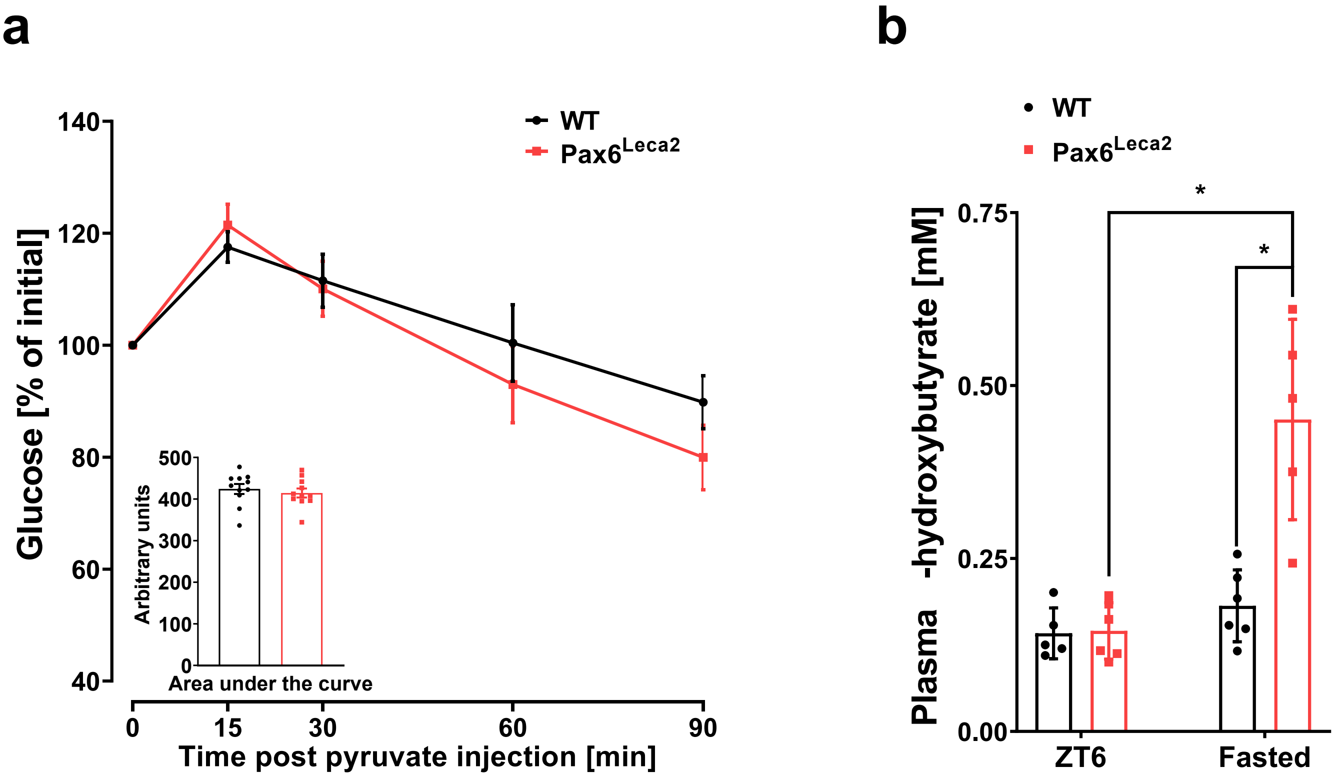

Supplementary fig 6 Short fasting period increases ketone bodies in Pax6^{Leca2} mice. a Pyruvate tolerance test. n=11. Error bars display ±s.e.m. **b** Plasma β-hydroxybutyrate levels at ZT6 (WT n=5, Leca2 n=6) and in 6-hour fasted mice (WT n=6, Leca2 n=5). *p<0.05 Welch's ANOVA followed by Dunnett's *post hoc* test. 12 week-old mice were used for both experiments. Error bars display ±s.d.

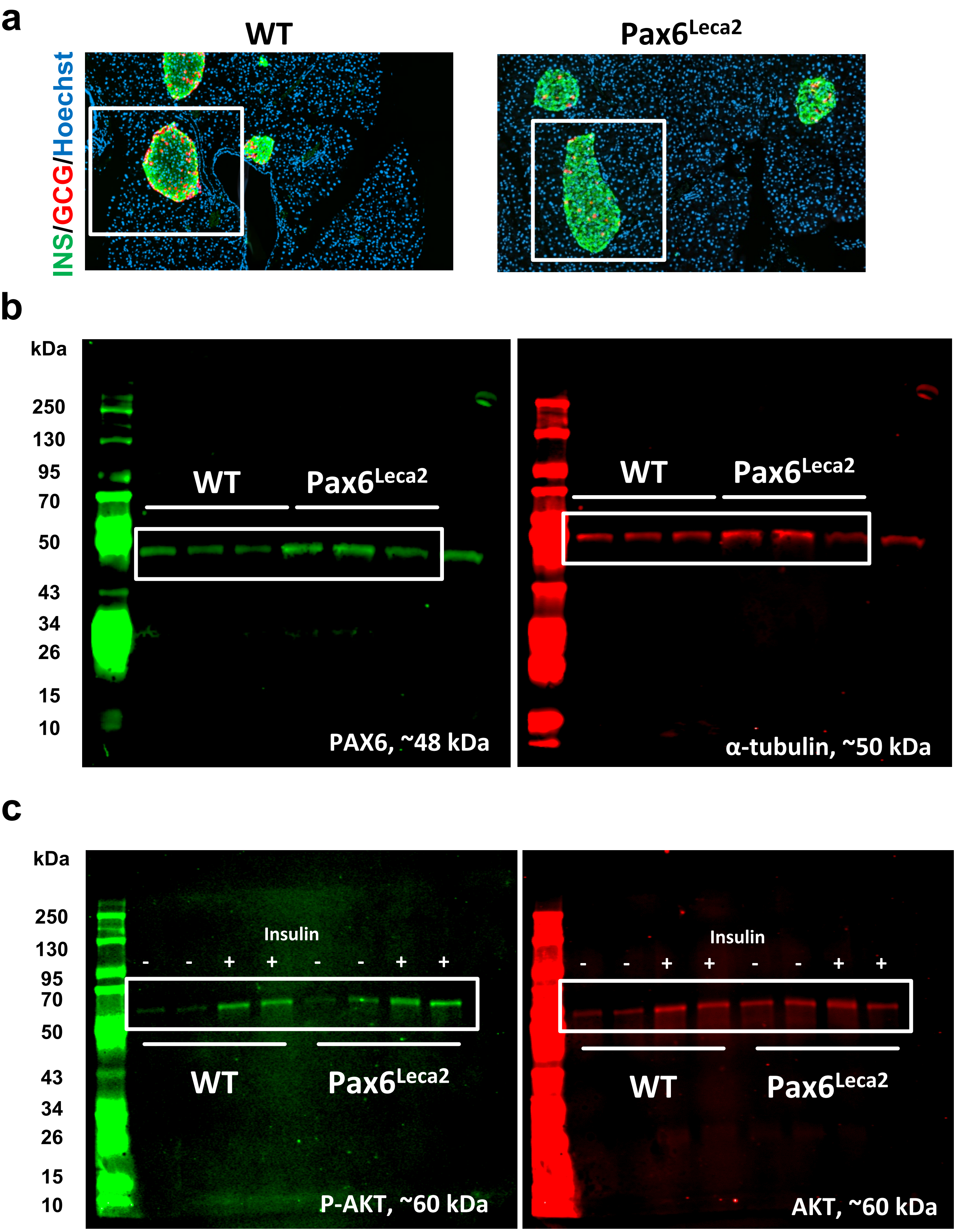

Supplementary fig 7 Source images. a Complete figure with marked cropped portions for Fig. 4e. **b** Complete figure with marked cropped portions for Supplementary fig. 4a. **c** Complete figure with marked cropped portions for Supplementary fig. 5d.
