# Supplementary material for: PAX6 mutation alters circadian rhythm and β cell function in mice without affecting glucose tolerance
Source: Commun Biol. 2020 Oct 30;3:628. doi: 10.1038/s42003-020-01337-x (PMC7599253; doi:10.1038/s42003-020-01337-x)
Supplement: Supplementary file 3 — Description of Additional Supplementary Files [file 42003_2020_1337_MOESM3_ESM.pdf]

Description of Additional Supplementary Files

File Name: Supplementary Data 1
Description: Set of 1440 significantly (FDR<10%) regulated genes between Pax6-Leca2 mice and WT mice (n=4). Genes were filtered for fold-change >1.5x and average expression >16 in at least one group.

File Name: Supplementary Data 2
Description: Sequences of primer pairs used in this study.

File Name: Supplementary Data 3
Description: Source data for all graphs in the main figures.
